# Teleoperated Grasping Using Data Gloves Based on Fuzzy Logic Controller

**DOI:** 10.3390/biomimetics9020116

**Published:** 2024-02-15

**Authors:** Chunxiao Lu, Lei Jin, Yufei Liu, Jianfeng Wang, Weihua Li

**Affiliations:** 1 School of Automotive Engineering, Harbin Institute of Technology (Weihai), Weihai 264209, China; 22s130337@stu.hit.edu.cn (C.L.); 18132835162@163.com (L.J.); wjfeee123@163.com (J.W.); 2 China North Vehicle Research Institute, Beijing 100072, China; liuyufei_noveri@outlook.com; 3 Yangtze River Delta HIT Robot Technology Research Institute, Wuhu 241060, China

**Keywords:** teleoperation, data gloves, fuzzy logic, robot control

## Abstract

Teleoperated robots have attracted significant interest in recent years, and data gloves are one of the commonly used devices for their operation. However, existing solutions still encounter two challenges: the ways in which data gloves capture human operational intentions and achieve accurate mapping. In order to address these challenges, we propose a novel teleoperation method using data gloves based on fuzzy logic controller. Firstly, the data are collected and normalized from the flex sensors on data gloves to identify human manipulation intentions. Then, a fuzzy logic controller is designed to convert finger flexion information into motion control commands for robot arms. Finally, experiments are conducted to demonstrate the effectiveness and precision of the proposed method.

## 1. Introduction

Recently, with human expansion into outer space and the deep sea, teleoperated robots capable of substituting humans in dangerous and harmful environments have become a popular technology [1,2,3]. Teleoperation is a human–robot collaboration system. Operators control distant robots through devices like joysticks and the robots leverage human cognition, creativity, and experience to complete tasks in complex environments. Human intervention in the system can compensate for deficiencies in robot control, sensing, and artificial intelligence, among others [4,5,6]. As of today, many scholars have researched teleoperation technology. Teleoperation robots are usually implemented using vision cameras and force feedback devices such as Geomagic Touch [7].

The vision-based teleoperation method uses visual cameras to capture real-time images of the operator’s gestures. These gesture images are then mapped into command information for the robot arm [8,9,10,11]. Du et al. [12] proposed a markerless hand tracking algorithm for real-time teleoperation of robot arms. They used depth images from Kinect to estimate the position and posture of the index finger and thumb in three-dimensional space. The calculated gestures controlled the posture of end effectors in real time. This method has been validated effectively through pick-and-place object experiments. Handa et al. [13] developed a depth camera-based teleoperation system called DexPilot, where two depth cameras visually enable experimenters to control the movements of robot arms and fingers barehandedly.

However, the vision-based teleoperation method needs to combine camera images to calculate the position and movement of human hands. This method needs to be conducted in a well-lit, obstacle-free environment. Thus, it has high requirements for the experimental environment and is limited by distance.

Robot teleoperation based on force feedback devices utilizes devices like Geomagic Touch to provide haptic feedback [14,15,16]. Operators transmit commands to the robot through the handle and simultaneously receive the interaction information from the robot’s interaction with the external environment. Zhu et al. [17] designed a dual manipulator robotic teleoperation system structure equipped with modular hybrid grippers. Its master end was composed of two Geomagic Touches and one leap motion controller, while the slave end encompassed two mechanical arms fitted with two grippers. The leap motion controller was tasked with tracking human hand movements. Asad et al. [18] presented a novel state convergence control algorithm for bilateral tele-operation of robotic systems, emphasizing a model in state space and a design procedure to compute control gains. In 2022, Asad et al. [19] proposed an extended composite state convergence scheme for multi-primary, multi-secondary teleoperation systems, incorporating a disturbance observer-based architecture to handle uncertainties. Yang et al. [20] introduced a synchronization control algorithm for a networked nonlinear master-slave teleoperation system, addressing operator fatigue in continuous operations.

However, force-feedback teleoperation based on Geomagic Touch requires professional equipment and operating skills. This makes it inconvenient to use, especially in scenarios such as assisting the elderly and disabled.

To overcome these issues, researchers began to focus on more natural, intuitive, and precise teleoperation methods. Data glove technology emerged, which uses built-in sensors to detect hand movement and posture information [21,22,23,24] and transmits these data to the robot arm system. By wearing a data glove, operators can manipulate mechanical arms in real time by changing hand movements. This offers higher operation precision and freedom of freedom, particularly during tasks such as grasping or placement, demonstrating the immense potential of data gloves [25,26,27,28].

Colasanto et al. [29] proposed a method using data gloves to capture finger motion information, mapping human hand movements onto robotic bionic hands. They developed a hybrid mapping combining joint space mapping and fuzzy-based posture mapping, which can achieve both joint mapping based on human hand movements and posture mapping for grasping tasks. Tyagi et al. [30] proposed using a fuzzy inference system to control the movement of an intelligent wheelchair. By wearing gloves equipped with flex sensors, they used their fingers to control wheelchair movement.

However, the above-mentioned data glove-based method faces the following problems: (1) The data glove-based teleoperation control system involves multi-joint motion control. Owing to the kinematic differences between human hands and robot arm systems, teleoperation cannot be simply mapped. (2) Due to varying human skeletal structures and different operator behavior habits, determining ways to process the sensor information from data gloves to accurately capture the manipulation intentions of different operators is key.

Inspired by the above literature review, a teleoperation method using data gloves based on fuzzy logic controller is proposed. Firstly, the human hand’s skeletal structure and movements are analyzed to obtain finger flexion information through flex sensor data from the data gloves. Subsequently, a fuzzy logic controller is designed to implement teleoperation control. The fuzzy logic controller captures human manipulation intentions from finger flexion information and sends motion control commands to the robot arm through fuzzy logic rules. Through wireless communication, real-time data transmission and operation are realized. Finally, experiments verify the effectiveness of teleoperation based on data gloves. The main contributions of this paper are summarized as follows:

- We propose a teleoperation method using data gloves and normalize the flex sensor data to identify human manipulation intentions.
- To achieve control of teleoperation, a fuzzy logic controller is designed. The control commands for the joint velocity of the robot arm are obtained through fuzzy logic rules based on finger flexion information.
- The experimental results indicated that compared to the joint velocity output of the PID controller, the ability of the data gloves to use a fuzzy logic algorithm has better non-linearity and enhances the stability of teleoperated robot arm control.

The remainder of this paper is organized as follows: Section 2 offers an overview of the system and the details of the proposed method. Section 3 presents the experimental evaluation results. Section 4 concludes the paper.

## 2. Method

### 2.1. System Overview

**Figure 1:**
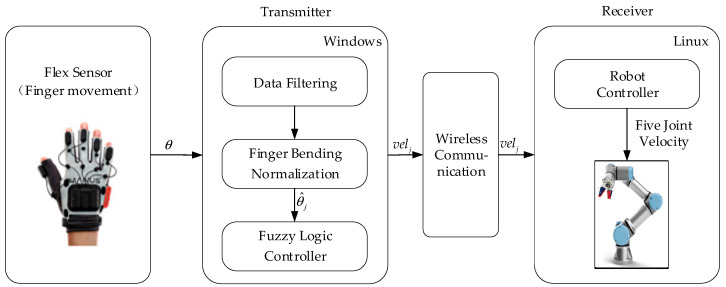
System overview.

As shown in Figure 1, the framework of the teleoperation system based on data gloves consists of two parts: Transmitter and Receiver. The operator wears data gloves and controls the movement of the robot arm in real time by flexing their fingers. The flex sensors and inertial sensors on the data gloves detect the degree of finger flexion. The Transmitter filters and standardizes information about finger flexion; the fuzzy logic controller processes input standard values of finger flexion degrees and outputs control commands for robot arms. The Receiver receives command information for controlling joint velocity on robot arms to control its motion state in real time. Data from flex sensors and robot control commands are transmitted wirelessly. The desired joint velocities given by the fuzzy controller are transmitted to the bottom controller for each joint embedded in the robot arm, which can track the desired commands based on the PID control law.

### 2.2. The Data Glove

#### 2.2.1. Hand Modeling

**Figure 2:**
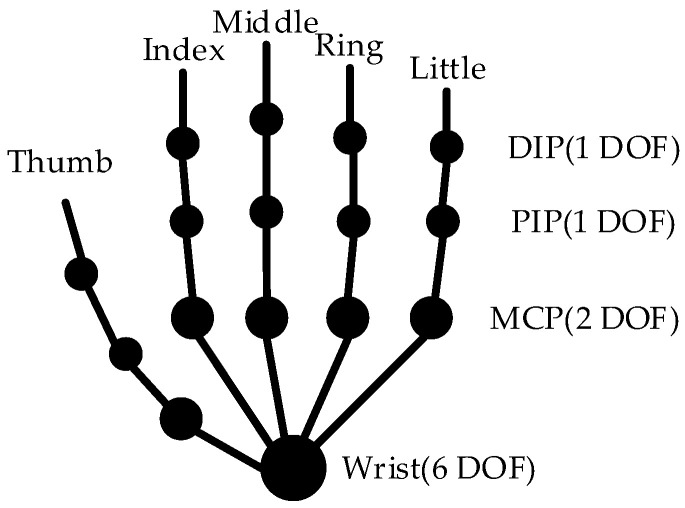
Human skeletal model.

Informed by anatomical analysis [31,32], the human hand skeletal model typically exhibits over 20 degrees of freedom, as shown in Figure 2. Fingers are composed of phalanges and finger joints, and they have flexion, extension, adduction, and abduction movement patterns. Each finger has four DOFs: the Meta-Carpophalangeal (MCP) joint has two DOFs, the Proximal–Interphalangeal (PIP) joint has one DOF, and the Distal Interphalangeals (DIP) joint has one DOF.

**Figure 3:**
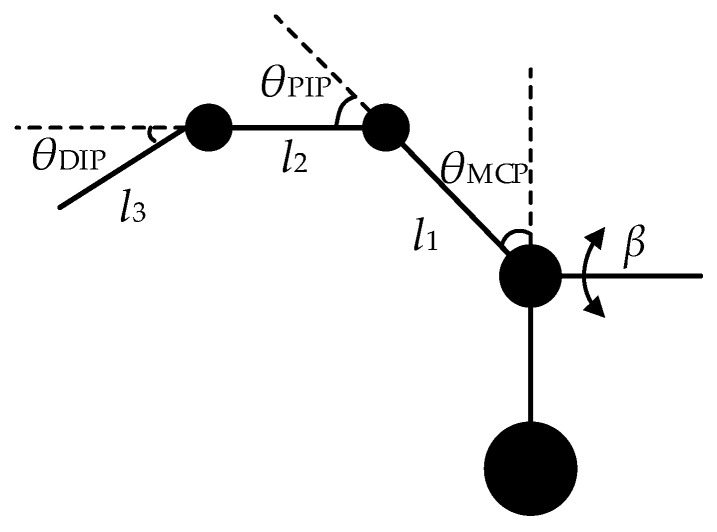
Simplified model of a human finger.

There are four methods for mapping human hand movements: fingertip mapping, joint mapping, key point mapping, and object-based mapping. Compared with direct manipulation in the end-effector space, joint angle mapping can not only regulate the configuration of the robot arm to ensure its safety, but also effectively complete the given tasks in the complex environments. We employ joint angle mapping. To streamline motion analysis, we simplify the human hand skeletal structure into a linkage structure, as depicted in Figure 3.

In the figure, *l*_1_ represents the length of the metacarpal bone, *l*_2_ represents the length of the proximal phalanx, *l*_3_ represents the length of the distal phalanx, *θ*_MCP_ represents the flexion angle of the Meta-Carpophalangeal joint, *θ*_PIP_ represents the flexion angle of the Proximal-Inter-phalangeal joint, *θ*_DIP_ represents the flexion angle of the Distal Inter-phalangeal joint and β indicates abduction and adduction angles at the Meta-Carpophalangeal joint.

#### 2.2.2. Data Filtering

**Figure 4:**
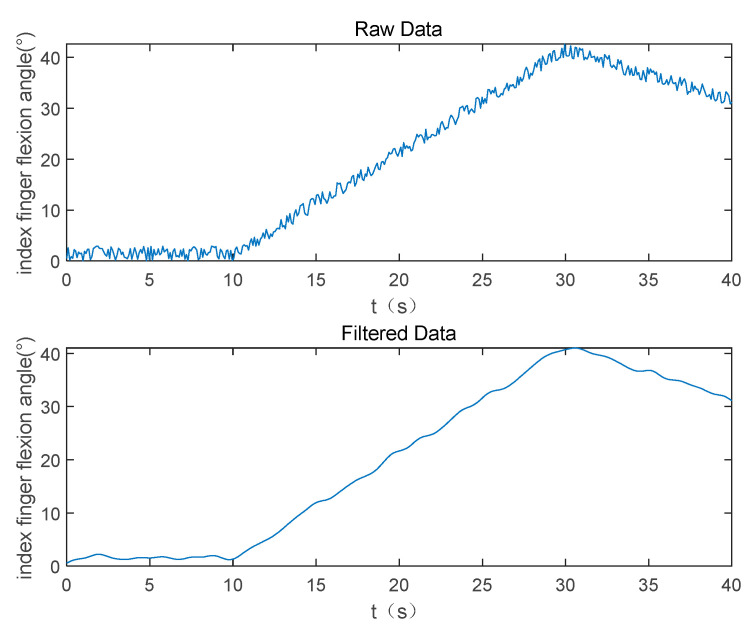
Filtered index finger flexion angle.

Due to environmental noise and uncertainty, instability and fluctuations of sensor measurements may occur. To eliminate interference from environmental noise, obtain more accurate and reliable hand bending angles, and enhance control stability, it is necessary to filter the data from the data glove. Figure 4 illustrates the raw data and filtered data of the index finger flexion angle. In this paper, a digital Butterworth low-pass filter is employed to eliminate high-frequency noise from the raw data, smoothing the data and reducing instability. The sensor sample rate of the data glove used in this paper is 90 Hz, with a cutoff frequency set at 5 Hz.

#### 2.2.3. Normalization

In the process of hand modeling, the flexion angle of each finger joint represents its current posture. Flex sensors on data gloves measure the degree of finger flexion. However, due to variations in human skeletal structures, finger sizes, and user habits, individuals might exhibit different physical ranges of finger flexion. To ensure the robustness of gesture mapping across diverse operators, this paper normalizes the degree of finger flexion within a specific range. This standardization facilitates the comparison of finger data across individuals and simplifies data complexity for more direct comparison and analysis.

(1) $${\hat{\theta }}_{j}=\frac{2\theta_{c}}{\theta_{j}}-1,\;j\in \left[\mathrm{MCP},\mathrm{PIP},\mathrm{DIP}\right],$$

where $\theta_{c}$ represents the current human finger joint flexion angle outputted by the flex sensor in the data glove, and $\theta_{j}$ represents the limit value of finger joint constraints. To eliminate unnatural gestures caused by sensor noise, upper and lower limits of finger joint motion are defined through joint constraints.

Human finger joint movement constraints [33] can be expressed as

(2) $$\left\{\begin{array}{l} \begin{array}{c} 0^{\circ }\leq \theta_{\mathrm{MCP}}\leq 90^{\circ }\\ 0^{\circ }\leq \theta_{\mathrm{PIP}}\leq 110^{\circ }\\ 0^{\circ }\leq \theta_{\mathrm{DIP}}\leq 90^{\circ } \end{array}\\ -15^{\circ }\leq \beta \leq 15^{\circ } \end{array}\right..$$

**Figure 5:**
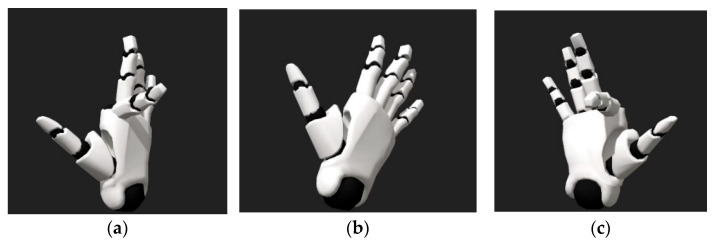
Schematic diagrams of three states: (**a**) Flexion standard value is −1; (**b**) Flexion standard value is 0; (**c**) Flexion standard value is 1.

The data gloves used in this study are equipped with five 2-DOF flex sensors and a 9-DOF IMU for each finger. The direction sensor has an accuracy of ±2.5 degrees, enabling precise tracking of finger movements with a signal delay of less than 5 ms, thus offering excellent real-time performance. The sensor frequency is 90 Hz, which is sufficient for tracking human finger movements. When the fingers bend towards the back of the hand, the standard value gradually changes from 0 to −1; when they bend towards the palm, it changes from 0 to 1; when fingers are straightened, their standard value is set at 0. Using the right index finger as an example, schematic diagrams of three states at standard values −1, 0 and 1 are shown in Figure 5.

The selection of gestures is guided by a thorough consideration of user intuitiveness. We conducted an analysis of common hand movements and gestures to identify that gestures were chosen to ensure users could easily associate them with the desired robot movements. The mapping for gestures to robotic commands is explained in Table 1 below. For the gesture depicted in Table 1, bending and straightening of the left ring finger controls the forward and reverse rotation of each joint.

### 2.3. Fuzzy Logic Controller

The dynamic characteristics of the teleoperated robot arm system based on data gloves are complex and difficult to represent with precise mathematical models. Fuzzy logic control introduces fuzzy logic into the control system, allowing the use of fuzzy, imprecise information to describe the behavior of teleoperated robot arm systems, thereby enhancing their nonlinearity. Therefore, this paper proposes a method based on fuzzy logic control to control remotely the robot arm.

**Figure 6:**
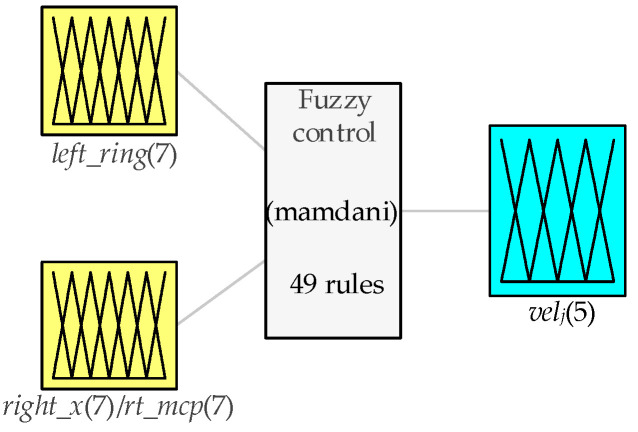
Design of fuzzy logic controller.

In Figure 6, *left_ring* represents the standard value of the left hand’s ring finger flexion; *right_x* represents the standard values of the right hand’s finger flexions, including the right hand’s thumb PIP joint (right_thumb_PIP), right hand’s index finger (right_index), right hand’s middle finger (right_middle), right hand’s ring finger (*right_ring*), and right hand’s little finger (right_little); rt_mcp represents the right hand’s thumb MCP joint; velj represents the joint velocity (rad/s) sent to Joint *j* of the robot arm, *j* ∈ [1,2,3,4,5].

Fuzzy logic controller is an intelligent control method that simulates artificial behavior based on human experience. Its process includes three steps: fuzzification, fuzzy inference, and defuzzification. This paper uses the standard value of left ring finger flexion and right finger flexion as two inputs for fuzzy logic controller. Joint velocities are considered as the output of the fuzzy controller instead of joint position, because the motion space of the fingers is relatively smaller compared to the motion space of the robot arm joints. Using joint position as the output may result in lower precision due to this kind of mapping.

For data gloves, mapping with the end-effector position of the robot arm is a challenging task byits finger joints. Unlike systems with clear end effectors in series or parallel structure of the general haptic devices, data gloves do not have a distinct end effector in the task space, making direct mapping complicated. When using data gloves for teleoperation, it becomes easier to conduct tasks by mapping the joint of the glove to the corresponding joint of the robot arm. Meanwhile, teleoperating the robot arm in the joint space can well optimize the joint configuration, contributing to safer and more complex movements.

**Figure 7:**
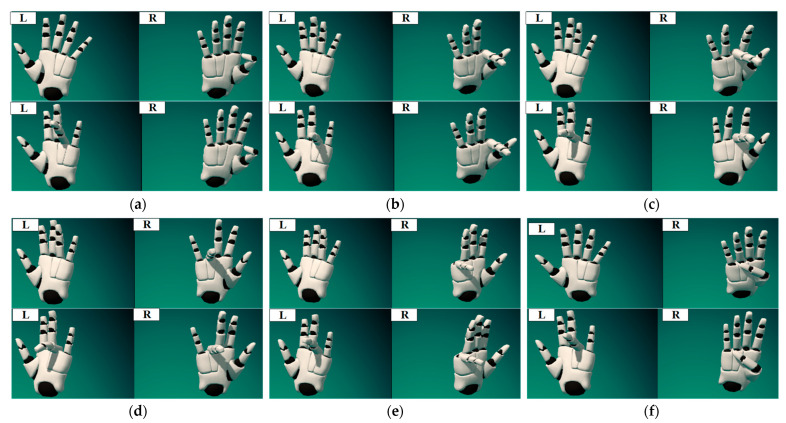
Gesture diagram of robot arm joint control: (**a**) Move joint 1 forward or reverse; (**b**) Move joint 2 forward or reverse. (**c**) Move joint 3 forward or reverse; (**d**) Move joint 4 forward or reverse; (**e**) Move joint 5 forward or reverse; (**f**) Move joint 6 forward or reverse.

As shown in Figure 7, the 1~6 joints of the UR3e robot arm are remotely controlled, respectively, through the PIP joint of the right thumb, right index finger, right middle finger, right ring finger, right little finger and MCP joint of the right thumb. The magnitude of the standardized finger flexion value indicates the magnitude of the robot arm joint velocity, and, according to left ring finger flexion standard value, changes the direction of rotation for robot arm joints. Based on the above analysis, fuzzy control rule base designed in this paper is shown in Table 2. The inputs are fuzzified into seven subsets: Very Small (VS), Small (S), Moderately Small (MS), Moderate (M), Moderately Large (ML), Large (L), Very Large (VL). The joint velocity of the mechanical arm is fuzzified into five subsets: NB, NS, ZE, PS, PB.

**Figure 8:**
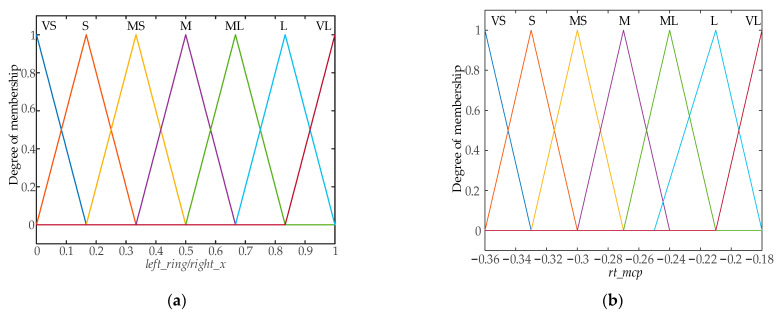
Input membership functions: (**a**) Membership function of left_ring/right_x; (**b**) Membership function of rt_mcp.

**Figure 9:**
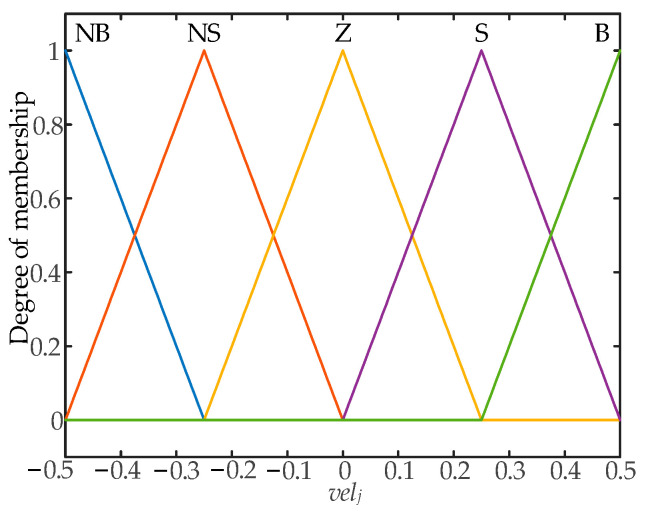
Input membership functions.

**Figure 10:**
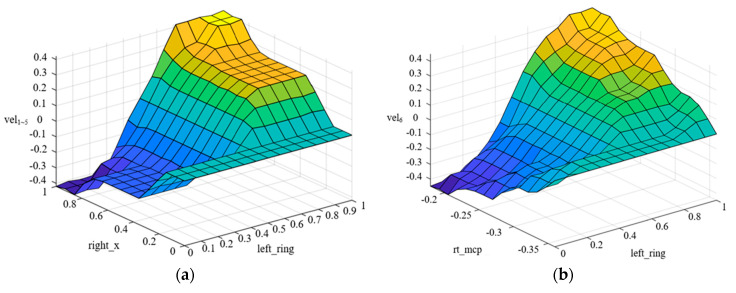
Fuzzy control surfaces: (**a**) Joint 1~5 velocity output surface; (**b**) Joint 6 velocity output surface.

A triangular membership function is used to implement the fuzzification of inputs and outputs in the fuzzy control system. The fuzzy control input membership function designed is shown in Figure 8, and the output membership function is shown in Figure 9. The fuzzy control surface is shown in Figure 10.

The above controller output value is defuzzified to derive an accurate inference result. This paper employs centroid-based defuzzification on robot arm joint velocity to procure precise output values [34].

(3) $$v_{j}=\frac{\int vel_{j}\mu_{vel_{j}}(vel_{j})dvel_{j}}{\int \mu_{vel_{j}}(vel_{j})dvel_{j}},$$

where *vel_j_* represents the fuzzy value of the joint velocity of the robot arm; *μ_velj_* represents the membership function value of the joint velocity of the robot arm; *v_j_* represents the precise value of joint velocity for robot arm.

## 3. Experiments

The experiments are conducted in two parts: the first experiment is carried out to test the effect of the fuzzy controller on the control of the robot arm joints, and the second experiment is conducted to observe the whole system’s performance in robot arm grasping.

### 3.1. Experimental Setup

**Figure 11:**
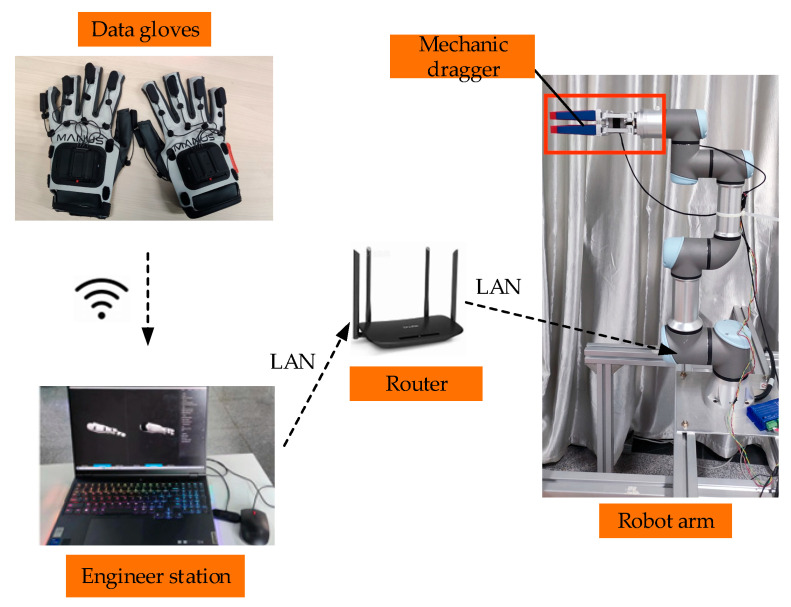
Experimental platform.

**Figure 12:**
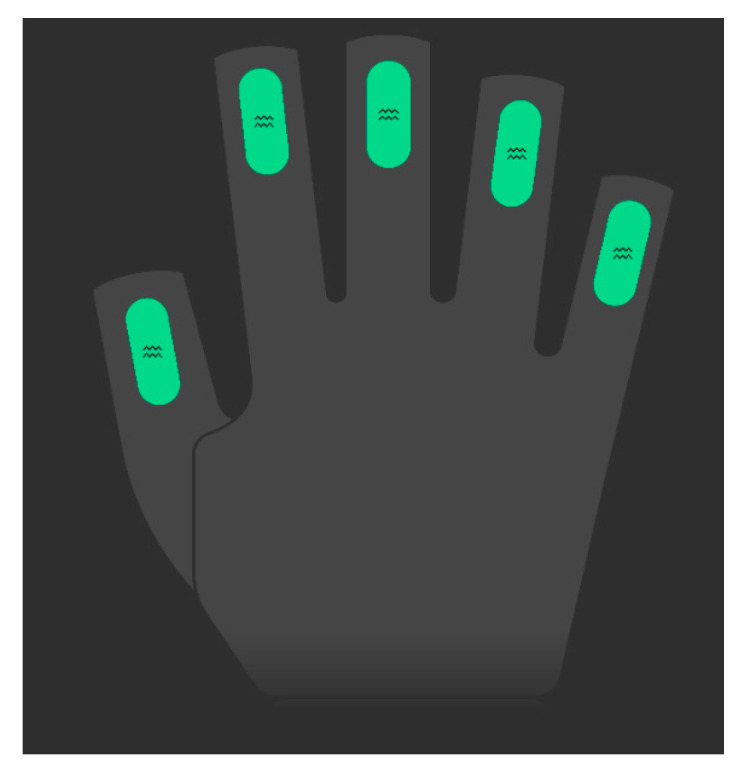
The flex sensors.

As shown in Figure 11, the experimental platform consists of Prime X Haptic VR data gloves from MANUS, a Lenovo Savior Y9000P laptop, Easy Gripper stepper motor mechanical claw, and UR3e robot arm with a force/torque sensor from Universal Robots company. The data glove is equipped with flex sensors and inertial sensors. The flex sensors can detect the degree of finger flexion, as shown in Figure 12. The experiment communicates between ROS and robot arm wirelessly, realizing cross-platform communication between Windows and Linux based on the distributed operating system. It achieves real-time data transmission between the data glove and the robot arm.

In our experimental setup, we utilize the wireless LAN (Local Area Network) to ensure the finger flexion data collected by the gloves could be transmitted to the controller on Linux. 

### 3.2. Fuzzy Logic Control Testing

In order to validate the effectiveness of the proposed method, we designed two teleoperation robot arm methods: one is based on a PID controller for remotely controlling the robot arm, and the other is based on a fuzzy logic controller for remotely controlling the robot arm. The UR3e robot arm’s Joints 1 to 6 are remotely manipulated through the PIP joint of the right thumb, right index finger, right middle finger, right ring finger, right little finger and MCP joint of the right thumb. Using one-dimensional velocity as an example, we conducted an experimental comparison between the two controllers.

By combining the bending of the left ring finger and the right fingers, remote control of Joints 1–5 of the robot arm was achieved. Specifically, the right thumb controlled the movement speed of Joint 1, the right index finger controlled the movement speed of Joint 2, the right middle finger controlled the movement speed of Joint 3, the right ring finger controlled the movement speed of Joint 4, and the right little controlled the movement speed of Joint 5. The bending and straightening of the left ring finger controlled the forward and reverse motion of each joint, thereby completing the control of the robot arm based on the data glove. The mapping of the standard values of bending in the fingers of the left and right hands to the joint velocities of the robot arm using a PID controller is as follows:

(4) $$vel_{j}=\left\{\begin{array}{l} right\_x-0.5,\;left\_ring<0.5\;\mathrm{and}\;0.5<right\_x\\ -(right\_x-0.5),\;0.5<left\_ring\;\mathrm{and}\;0.5<right\_x\\ 0,\;\mathrm{else} \end{array}\right.$$

where *left_ring* represents the standard value of left hand’s ring finger flexion; *right_x* represents the standard values of right hand’s finger flexions, including right thumb (right_thumb), right index finger (right_index), right middle finger (right_middle), right ring finger (*right_ring*), and right little finger (right_little); velj represents the joint velocity (rad/s) sent to Joint *j* of the robot arm, *j* ∈ [1,2,3,4,5].

**Figure 13:**
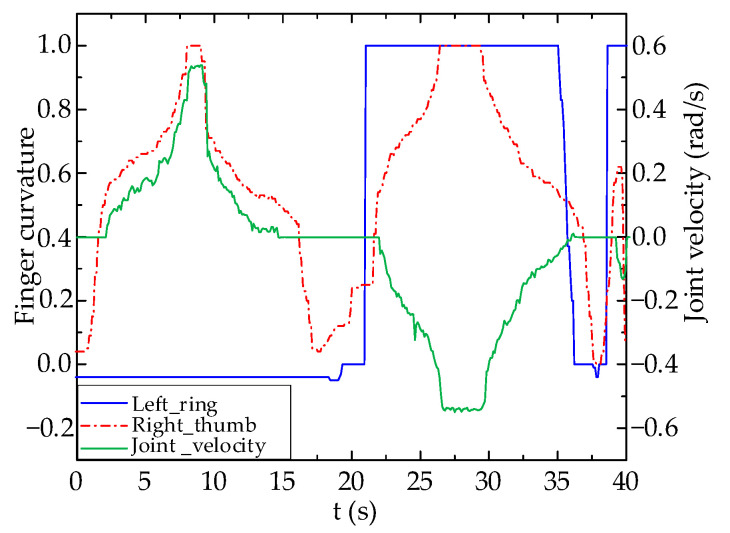
PID Controller Test Results.

**Figure 14:**
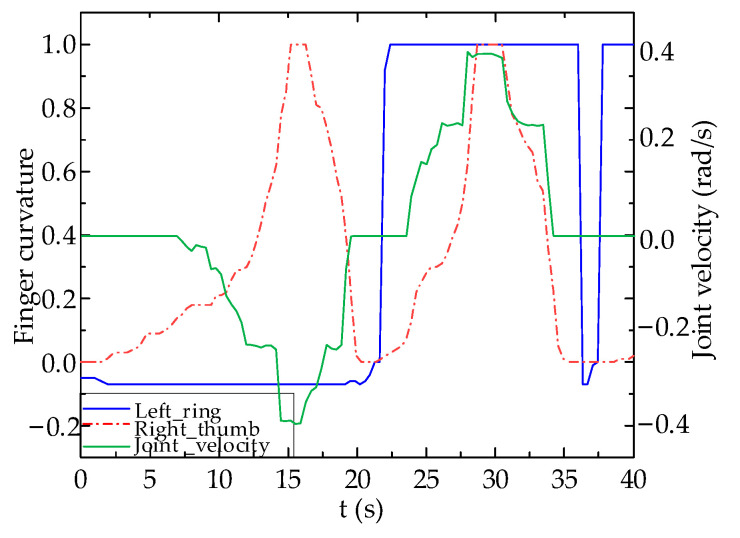
Fuzzy Logic Controller Test Results.

The experimental results are shown in Figure 13 and Figure 14. It can be seen that when left hand’s ring finger flexion standard value is less than 0.5 and right hand’s thumb flexion standard value gradually increases from a smaller value to approach 0, joint velocity of Joint 1 of robot arm gradually increases when right hand’s thumb flexion standard value is greater than 0.5 with movement direction towards left. When the right hand’s ring finger flexion standard value is less than 0.5, the velocity of Joint 1 remains at zero, thus indicating no movement. The movement of the robot arm matches the control expectations.

From Figure 14, it appears that compared to the joint velocity output of a PID controller, the data glove’s ability to control the robot arm using a fuzzy logic algorithm leads to better results than that of a PID controller. This is shown by its sensitivity to finger flexion changes. Moreover, its higher non-linearity prevents abrupt changes in joint velocity due to abnormal fluctuations in the data glove’s control values, enhancing the stability of teleoperated robot arm control. According to the experimental results, the measured delay time of the robot arm control system using a fuzzy controller is less than 0.05 s.

**Figure 15:**
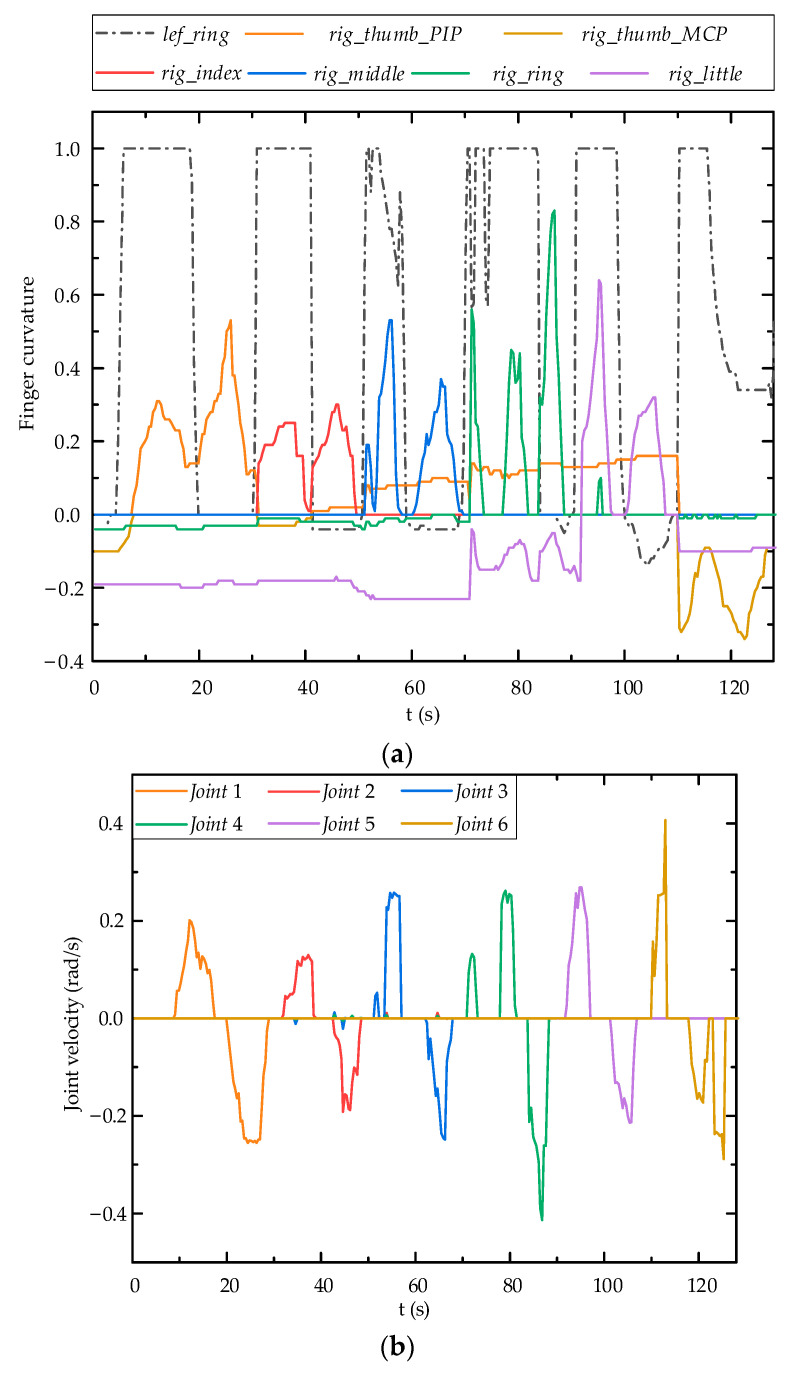
Motion test results using fuzzy logic controller: (**a**) Finger flexion standard value; (**b**) Joint velocity of robot arm.

In order to validate the teleoperation of the robot arm predicated on fuzzy logic control, the left ring finger governs the robot arm’s rotational direction, while the right hand independently controls the movement velocity of the robot arm’s joints. The standard values of each finger’s flexion and the robot arm’s joint velocity are shown in Figure 15. It can be seen from Figure 15 that the joint motion of the robot arms can follow the flexion motion of the fingers.

### 3.3. Teleoperated Grasping Testing

**Figure 16:**
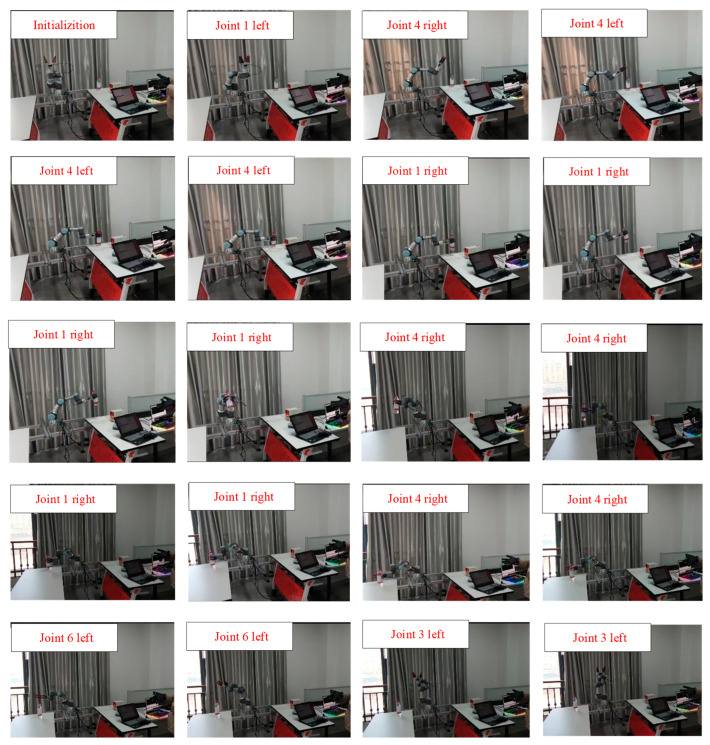
Robot arm grasping experiment.

Figure 16 shows an object grasping experiment designed to substantiate the comprehensive functionality of the remote operation system in this study. The experimental procedure comprises the following steps: (1) Moving to the initial position. (2) Grasping the object. (3) Shifting to the target position. (4) Releasing the object. (5) Reverting to the initial position.

**Figure 17:**
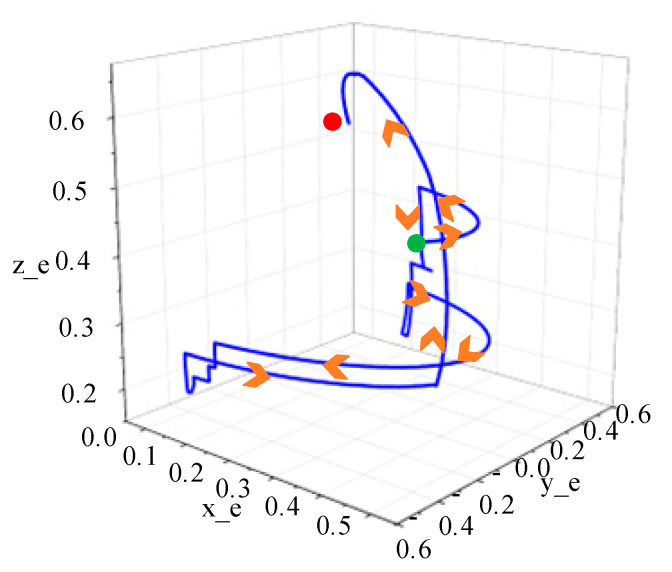
Robot arm end trajectory.

In the experiment of remotely operating a robot arm to grasp objects, the robot arm first rotates left to above the object. During its descent, it makes fine adjustments based on the relative position between the object and end effector of robot arm, ensuring that the end effector can smoothly grab the object. After grasping the object, the end effector of the robot arm rises to a certain height, then turns right until it reaches above the designated location. Then, the end effector slowly descends until the object completely touches table surface. Finally, the robot arm lifts up, completing the experiment of remotely operating a robot arm to grab an object. The position of the end effector is shown in Figure 17. The green point in Figure 17 represents the starting position of robot arm’s endpoint and the red point represents the final position.

**Figure 18:**
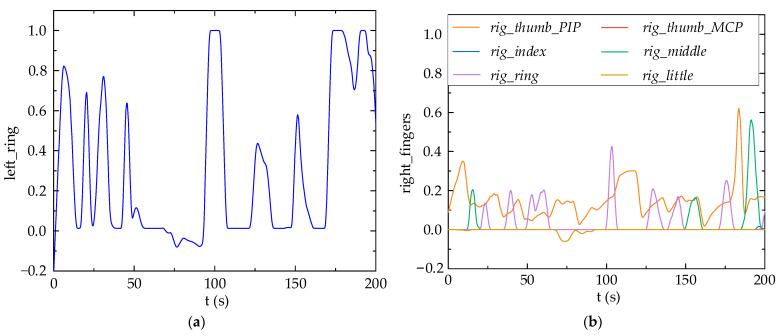
Glove data: (**a**) Finger curvature of the left ring; (**b**) Finger curvature of the right hand.

Figure 18 shows the information of the data gloves for both hands. During this process, the MCP joints of right index finger, right little finger, and right thumb always maintain a standard flexion value of zero. The movements of the robot arm mainly depend on Joint 1, Joint 3, and Joint 4, while Joints 2, 5, and 6 remain relatively stationary.

**Figure 19:**
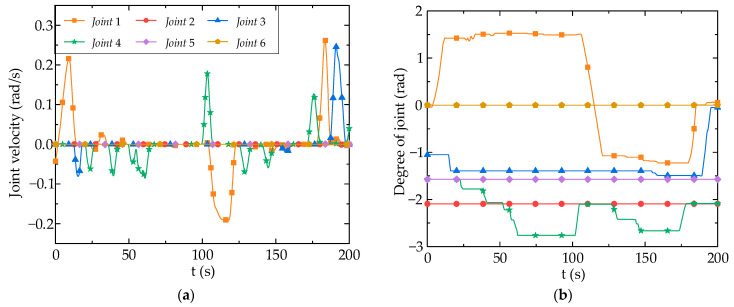
Robot arm joint velocity and position: (**a**) Robot arm joint velocity; (**b**) Robot arm joint position.

In the aforementioned movements, the joint velocity and joint position of the robot arm are shown in Figure 19. The change range of the joint position of robot arm is large, but the motion curve is relatively smooth, without abnormal jitter and jump, indicating that the fuzzy controller proposed in this paper is quite stable and reliable. The joint velocity of robot arm can follow finger flexion movement, change rotation direction according to changes in standard flexion value of left ring finger, and balance flexibility and stability at same time. According to the experimental results, using data gloves for teleoperation based on fuzzy logic controller produces higher non-linearity, which is sensitive to the finger flexion changes. The measured delay time of the control system using a fuzzy controller is less than 0.05 s due to the communication delay.

## 4. Conclusions

In this paper, a data glove-based teleoperation using fuzzy logic controller method was proposed and validated. We collected hand posture through data gloves and standardized the data to interpret human operational intentions. A fuzzy logic controller was designed to achieve precise control of teleoperation. The experimental results indicated that compared to the joint velocity output of the PID controller, the ability of the data glove to use a fuzzy logic algorithm for mobile robot control leads to better results. This is shown by its sensitivity to finger flexion changes. Moreover, its higher non-linearity prevents abrupt changes in joint velocity due to abnormal fluctuations in the data glove’s control values, enhancing the stability of teleoperated robot arm control.

To further improve the performance of the proposed teleoperation method, our future work will develop a more complete teleoperation system and consider the case of a robot interacting with the external environment.

## Figures and Tables

**Table 1:** The mapping for Gestures to Robotic.

Manipulator Action	Gesture
Return to position	No finger bend
Move robotic joint 1	Bend left ring finger (left_ring) and bend right thumb PIP joint (right_x)
Move robotic joint 2	Bend left ring finger (left_ring) and bend right index fin-ger (right_x)
Move robotic joint 3	Bend left ring finger (left_ring) and bend right middle fin-ger (right_x)
Move robotic joint 4	Bend left ring finger (left_ring) and bend right ring fin-ger (right_x)
Move robotic joint 5	Bend left ring finger (left_ring) and bend right little fin-ger (right_x)
Move robotic joint 6	Bend left ring finger (left_ring) and bend right thumb MCP joint (rt_mcp)

**Table 2:** Fuzzy control rule table.

Joint Velocity (vel_j_)		Left_Ring						
		VS	S	MS	M	ML	L	VL
right fingers’ flexion standard value	VS	Z	Z	Z	Z	Z	Z	Z
	S	Z	Z	Z	Z	Z	Z	Z
	MS	NS	NS	NS	Z	S	S	S
	M	NS	NS	NS	Z	S	S	S
	ML	NS	NS	NS	Z	S	S	S
	L	NB	NB	NS	Z	S	PB	PB
	VL	NB	NB	NB	Z	PB	PB	PB

## Data Availability

The data that support the findings of this study are available from the corresponding author upon reasonable request.

## References

[1] Batty T., Ehrampoosh A., Shirinzadeh B., Zhong Y., Smith J. (2022). A Transparent Teleoperated Robotic Surgical System with Predictive Haptic Feedback and Force Modelling. Sensors.

[2] Doisy G., Ronen A., Edan Y. (2017). Comparison of Three Different Techniques for Camera and Motion Control of a Teleoperated Robot. Appl. Ergon..

[3] Shiroma N., Miyauchi R., Matsuno F. (2008). Mobile Robot Teleoperation through Virtual Robot. Proceedings of the RO-MAN 2008-The 17th IEEE International Symposium on Robot and Human Interactive Communication.

[4] Zou R., Liu Y., Li Y., Chu G., Zhao J., Cai H. (2023). A Novel Human Intention Prediction Approach Based on Fuzzy Rules through Wearable Sensing in Human–Robot Handover. Biomimetics.

[5] Bauer A., Wollherr D., Buss M. (2008). Human–Robot collaboration: A survey. Int. J. Hum. Robot..

[6] Xia Y., Li W., Duan S., Lei W., Wu J. (2022). Low-Cost, Light-Weight Scalable Soft Data Glove for VR Applications. Proceedings of the 2022 5th International Conference on Circuits, Systems and Simulation (ICCSS).

[7] Alves de Oliveira T.E., Cretu A.-M., Petriu E.M. (2017). Multimodal Bio-Inspired Tactile Sensing Module. IEEE Sens. J..

[8] Li S., Ma X., Liang H., Görner M., Ruppel P., Fang B., Sun F., Zhang J. Vision-Based Teleoperation of Shadow Dexterous Hand Using End-to-End Deep Neural Network. Proceedings of the 2019 International Conference on Robotics and Automation (ICRA).

[9] Li S., Jiang J., Ruppel P., Liang H., Ma X., Hendrich N., Sun F., Zhang J. (2020). A Mobile Robot Hand-Arm Teleoperation System by Vision and Imu. Proceedings of the 2020 IEEE/RSJ International Conference on Intelligent Robots and Systems (IROS).

[10] Gomez-Donoso F., Orts-Escolano S., Cazorla M. (2019). Accurate and Efficient 3D Hand Pose Regression for Robot Hand Teleoperation Using a Monocular RGB Camera. Expert Syst. Appl..

[11] Yang W., Paxton C., Cakmak M., Fox D. Human Grasp Classification for Reactive Human-to-Robot Handovers. Proceedings of the 2020 IEEE/RSJ International Conference on Intelligent Robots and Systems (IROS).

[12] Du G., Zhang P., Mai J., Li Z. (2012). Markerless Kinect-Based Hand Tracking for Robot Teleoperation. Int. J. Adv. Robot. Syst..

[13] Handa A., Van Wyk K., Yang W., Liang J., Chao Y.-W., Wan Q., Birchfield S., Ratliff N., Fox D. DexPilot: Vision-Based Teleoperation of Dexterous Robotic Hand-Arm System. Proceedings of the 2020 IEEE International Conference on Robotics and Automation (ICRA).

[14] Chotiprayanakul P., Liu D.K. (2009). Workspace Mapping and Force Control for Small Haptic Device Based Robot Teleoperation. Proceedings of the 2009 International Conference on Information and Automation.

[15] Farkhatdinov I., Ryu J.-H., An J. (2010). A Preliminary Experimental Study on Haptic Teleoperation of Mobile Robot with Variable Force Feedback Gain. Proceedings of the 2010 IEEE Haptics Symposium.

[16] Tang Y., Liu S., Deng Y., Zhang Y., Yin L., Zheng W. (2020). Construction of Force Haptic Reappearance System Based on Geomagic Touch Haptic Device. Comput. Methods Programs Biomed..

[17] Zhu G., Xiao X., Li C., Ma J., Ponraj G., Prituja A.V., Ren H. (2020). A Bimanual Robotic Teleoperation Architecture with Anthropomorphic Hybrid Grippers for Unstructured Manipulation Tasks. Appl. Sci..

[18] Asad M.U., Farooq U., Gu J., Abbas G., Liu R., Balas V.E. (2019). A Composite State Convergence Scheme for Bilateral Teleoperation Systems. IEEE/CAA J. Autom. Sin..

[19] Asad M.U., Gu J., Farooq U., Balas V.E., Balas M.M., Abbas G. (2022). An Improved Composite State Convergence Scheme with Disturbance Compensation for Multilateral Teleoperation Systems. Stud. Inform Control.

[20] Yang Y., Jiang H., Hua C., Li J. (2024). Practical Preassigned Fixed-Time Fuzzy Control for Teleoperation System under Scheduled Shared-Control Framework. IEEE Trans. Fuzzy Syst..

[21] Fang B., Guo D., Sun F., Liu H., Wu Y. (2015). A Robotic Hand-Arm Teleoperation System Using Human Arm/Hand with a Novel Data Glove. Proceedings of the 2015 IEEE International Conference on Robotics and Biomimetics (ROBIO).

[22] Caeiro-Rodríguez M., Otero-González I., Mikic-Fonte F.A., Llamas-Nistal M. (2021). A Systematic Review of Commercial Smart Gloves: Current Status and Applications. Sensors.

[23] Basjaruddin N.C., Sutjiredjeki E., Akbar H.W.C. (2019). Developing an Electronic Glove Based on Fuzzy Logic for Mobile Robot Control. J. Intell. Fuzzy Syst..

[24] Rodriguez L., Przedworska Z., Obidat O., Parron J., Wang W. (2022). Development and Implementation of an AI-Embedded and ROS-Compatible Smart Glove System in Human-Robot Interaction. Proceedings of the 2022 IEEE 19th International Conference on Mobile Ad Hoc and Smart Systems (MASS).

[25] Ali M., Malik A., Yusof Z.M., Kushairy A.K., Zaharah H.F., Ismail A. (2015). Development of Smart Glove System for Therapy Treatment. Proceedings of the 2015 International Conference on BioSignal Analysis, Processing and Systems (ICBAPS).

[26] Li F., Chen J., Zhou Z., Xie J., Gao Z., Xiao Y., Dai P., Xu C., Wang X., Zhou Y. (2023). Lightweight Soft Robotic Glove with Whole-Hand Finger Motion Tracking for Hand Rehabilitation in Virtual Reality. Biomimetics.

[27] Zhu Q., Da Fonseca V.P., Lima B.M.R., Welyhorsky M., Goubran M., De Oliveira T.E.A., Petriu E.M. (2020). Teleoperated Grasping Using a Robotic Hand and a Haptic-Feedback Data Glove. Proceedings of the 2020 IEEE International Systems Conference (SysCon).

[28] Dhepekar P., Adhav Y.G. (2016). Wireless Robotic Hand for Remote Operations Using Flex Sensor. Proceedings of the 2016 International Conference on Automatic Control and Dynamic Optimization Techniques (ICACDOT).

[29] Colasanto L., Suarez R., Rosell J. (2013). Hybrid Mapping for the Assistance of Teleoperated Grasping Tasks. IEEE Trans. Syst. Man Cybern. Syst..

[30] Tyagi V., Gupta N.K., Tyagi P.K. (2013). Smart Wheelchair Using Fuzzy Inference System. Proceedings of the 2013 IEEE Global Humanitarian Technology Conference: South Asia Satellite (GHTC-SAS).

[31] ElKoura G., Singh K. Handrix: Animating the Human Hand. Proceedings of the 2003 ACM SIGGRAPH/Eurographics Symposium on Computer Animation.

[32] Li K., Chen I.-M., Yeo S.H., Lim C.K. (2011). Development of Finger-Motion Capturing Device Based on Optical Linear Encoder. J. Rehabil. Res. Dev..

[33] Lin J., Wu Y., Huang T.S. (2000). Modeling the Constraints of Human Hand Motion. Proceedings of the Proceedings Workshop on Human Motion.

[34] Tanaka K., Sugeno M. (1992). Stability Analysis and Design of Fuzzy Control Systems. Fuzzy Sets Syst..

